# Differential expression of miRNAs in the body wall of the sea cucumber *Apostichopus japonicus* under heat stress

**DOI:** 10.3389/fphys.2022.929094

**Published:** 2022-07-21

**Authors:** Mengyang Chang, Bin Li, Meijie Liao, Xiaojun Rong, Yingeng Wang, Jinjin Wang, Yongxiang Yu, Zheng Zhang, Chunyuan Wang

**Affiliations:** ^1^ Key Laboratory of Sustainable and Development of Marine Fisheries, Ministry of Agriculture and Rural Affairs, Yellow Sea Fisheries Research Institute, Chinese Academy of Fishery Sciences, Qingdao, China; ^2^ College of Fishers and Life Science, Shanghai Ocean University, Shanghai, China; ^3^ Laboratory for Marine Fisheries Science and Food Production Processes, Qingdao National Laboratory for Marine Science and Technology, Qingdao, China

**Keywords:** *Apostichopus japonicus*, microRNA, heat stress, stress response, miRNA-mRNA network

## Abstract

MicroRNAs, as one of the post-transcriptional regulation of genes, play an important role in the development process, cell differentiation and immune defense. The sea cucumber *Apostichopus japonicus* is an important cold-water species, known for its excellent nutritional and economic value, which usually encounters heat stress that affects its growth and leads to significant economic losses. However, there are few studies about the effect of miRNAs on heat stress in sea cucumbers. In this study, high-throughput sequencing was used to analyze miRNA expression in the body wall of sea cucumber between the control group (CS) and the heat stress group (HS). A total of 403 known miRNAs and 75 novel miRNAs were identified, of which 13 miRNAs were identified as significantly differentially expressed miRNAs (DEMs) in response to heat stress. A total of 16,563 target genes of DEMs were predicted, and 101 inversely correlated target genes that were potentially regulated by miRNAs in response to heat stress of sea cucumbers were obtained. Based on these results, miRNA-mRNA regulatory networks were constructed. The expression results of high-throughput sequencing were validated in nine DEMs and four differentially expressed genes (DEGs) by quantitative real-time polymerase chain reaction (RT-qPCR). Moreover, pathway enrichment of target genes suggested that several important regulatory pathways may play an important role in the heat stress process of sea cucumber, including ubiquitin-mediated proteolysis, notch single pathway and endocytosis. These results will provide basic data for future studies in miRNA regulation and molecular adaptive mechanisms of sea cucumbers under heat stress.

## Introduction

The sea cucumber (*Apostichopus japonicus*) is widely distributed along the northwest Pacific coast at 35∼44°N and has been utilized as an important marine economic resource for its nutritional and medicinal value (Chang and Loscalzo, 2010; Wang et al., 2015). The total amount of sea cucumber cultivation in China has continuously increased and the cultivation area has been expanding since 2003. In 2020, the annual production of sea cucumber cultivation reached 200,000 t with an increase of 14.48% over the previous year, and the production value reached 50 billion US dollars (Ministry of Agriculture and Rural Affairs, 2021). The sea cucumber aquaculture industry has suffered great loss, with the global warming and the frequent occurrence of high temperatures in summer. Heat stress was reported to impose many negative effects on the growth, metabolism, and reproduction of aquatic farmed animals. Therefore, understanding the molecular mechanism of miRNA under heat stress would help to reduce the damage caused by heat stress on sea cucumbers and breed new varieties with high temperature resistance.

As a typical temperate species, the sea cucumber was greatly affected by water temperature, not only on its physiological activities, but also on feeding and metabolism (Zhao et al., 2015; Li et al., 2016a). The high temperature was shown to reduce the activity and food intake of sea cucumber, disorder the metabolism of free radicals in the body and even lead to disease and death (Wang et al., 2011; Li et al., 2012). It was showed that the total number of coelomic cells, phagocytosis rate and lysozyme activity in the coelomic fluid of *A. japonicus* was decreased to a certain extent after entering summer dormancy (Shao et al., 2016). The research on the effects of temperature changes on the physiology, growth and gene expression of sea cucumbers has gotten a lot of attention. Zhang et al. (2015) found that increasing temperature can affect the DNA methylation level of *A. japonicus*, and then change the expression of the heat shock protein gene. The methylation sites of intestinal, respiratory tree and gonadal tissues in sea cucumber were mainly distributed in the gene functional regions, and the methylation sites were mainly of CG type (Li et al., 2018). Under heat stress at 26°C, the methylation level of intestinal genome in sea cucumber increased, while at 32°C, the methylation level decreased (Wen et al., 2021). At present, the research on the regulation mechanism of sea cucumber under heat stress focuses on the epigenetic and transcriptional regulation, but there are few studies on the post-transcriptional regulation.

MicroRNAs (miRNAs) are endogenous noncoding small RNAs with 22 nt, which regulate post-transcriptional gene expression by binding to the 3′ untranslated region (UTR) of target genes (Bartel, 2004; Pedersen et al., 2007). Numerous studies have reported that miRNAs can provide reversible gene silencing mechanisms during animal aestivation and hibernation, thus miRNAs can be involved in cellular adaptation to specific demands under stressful conditions (Jones-Rhoades and Bartel, 2004). It has been reported that miRNA played a key role in the response to heat stress of plants and animals (Jones-Rhoades and Bartel, 2004). In rainbow trout, some miRNAs, including ssa-miR-301a-3p, ssa-miR-30a-5p and ssa-miR-30a-5p, can regulate the key changes of cells under high temperature stress (Ma et al., 2019). In *Litopenaeus vannamei*, 41 differentially expressed miRNAs related to heat stress were selected, such as lva-miR-92b, lva-miR-317 and lva-miR-184 (Boonchuen et al., 2020). Recently, Li et al. (2016b) and Zhou et al. (2018) found that some miRNAs including miR-184 and miR-2004 played a key role in the coelomic fluid of sea cucumber in response to heat stress. However, it was largely unknown for the miRNA regulation in the body wall of diseased sea cucumber in response to heat stress.

The purpose of this study was to identify known miRNAs and novel miRNAs from the body wall of sea cucumber high-throughput sequencing analysis, as well as to investigate the regulation mechanism between miRNAs and their target genes in sea cucumber after heat treatment. Our findings will be useful in further studies of sea cucumber biomarkers and will provide basic data for future studies on miRNA regulation and sea cucumber molecular adaptive mechanisms under heat stress.

## Materials and methods

### Experimental design and tissue collection

Sea cucumbers (body weight 50.0 ± 2.0 g) of appr. 1 year old were collected from Qingdao Ruizi Group Co., Ltd., in Shandong Province, China. The sea cucumbers were transported to our lab in Qingdao and acclimated in aerated indoor tanks for 5 days. During the experiment, the temperature and salinity of sea water were around 15°C and 30‰, respectively. The sea cucumbers were fed with a regular compound feed, and one third of seawater was changed daily.

When acclimation finished, a total of 30 sea cucumbers were selected and randomly divided into two groups. For the control group (CS), the culture temperature was continued to maintain at 15°C. According to previous studies of our lab, the median lethal temperature (LT_50_) to sea cucumber was 32°C (Zhang et al., 2022). Therefore, the temperature for the heat stress group (HS) was set to 32°C in the present study. Heaters were used to give thermal stress to the sea cucumbers. The temperature system was continuously increased at a heating rate of 2°C/24 h. The moment when the water temperature reached 32°C was regarded as the initial time. In the subsequent experiment, the water temperature was maintained at 32°C ± 0.5°C. On the third day of the experiment, three sea cucumbers in the CS group and three skin ulceration syndrome individuals in the HS group were randomly selected. The body wall of six sea cucumbers were immediately sampled and frozen in liquid nitrogen, since the body wall was the target organ of skin ulceration syndrome. All the samples were stored at −80°C for miRNA sequencing analysis.

### RNA extraction and processing

Total RNA was extracted from each sample using the Animal Tissue RNA Purification Kit (LC Sciences, Houston, TX, United States) according to the manufacturer’s instructions. The quality of total RNA was checked in Bioanalyzer 2100 (Agilent, Santa Clara, CA, United States) with RNA integrity number >7.0.

Small RNA library was constructed according to the protocol of TruSeq Small RNA Sample Prep Kits (Illumina, San Diego, CA, United States). RNAs in the 16–30 nt size range were purified from a 15% polyacrylamide gel, then sequentially ligated to 5′ and 3′ adapters. Reverse transcription was subsequently performed by polymerase chain reaction (PCR) amplification. The purified PCR products were sequenced by Illumina Hiseq2500 (LC-BIO, Hangzhou, China).

### Sequence data analysis

Raw reads were subjected to an in-house program, ACGT101-miR (LC Sciences, Houston, TX, United States) to remove adapter dimers, junk, low complexity, and clean reads ranging from 16 to 30 nt in length were obtained. Sequence matching non-coding RNAs, including rRNA, tRNA, small nuclear RNA (snRNA), and small nucleolus RNA (snoRNA), were mapped to Repbase database (http://www.girinst.org) and Rfam database (http://rfam.xfam.org). The unique small RNA sequences were aligned against the miRBase V22.0 (http://www.mirbase.org/blog) to select the known miRNAs and novel miRNAs and at most one mismatch inside of the sequence was allowed in the alignment. The Deuterostoma species in the miRBase were used as host species and the priority rank of these species in sequence alignment was listed in Supplementary Table S1. Then, the sequence was mapped to the sea cucumber genome (assembled by our lab, unpublished) by Bowtie 1 (Berthelot et al., 2014) to determine the genomic locations of known miRNAs and the flank sequences of novel miRNAs. The hairpin structures of the novel miRNAs were predicated from the flank 80 nt sequences using miRDeep2 (Friedlnder et al., 2012). Principal component analysis (PCA) was performed on the valid data using the vegan R package.

TPM (millions of transcripts) normalization was used for miRNA expression analysis. Differential expression of miRNAs was selected using DEGseq (http://www.bioconductor.org/packages/release/bioc/html/DEG-seq.html). Fold-change and *p*-values were calculated from the normalized expression referring to the methods in published studies (Song et al., 2017). |log_2_(Foldchange)| > 1 and *p*-value lower than 0.05 was considered as significantly different expression miRNAs (DEMs).

### Target gene prediction and function analysis

MiRanda (http://www.microrna.org/) and TargetScan (http://www.targetscan.org/vert_80/) were used to predict the target genes of known miRNAs and novel miRNAs. Target genes with a context score percentile <50 were removed from the TargetScan, and target genes with maximum free energy (Max Energy) >−10 were removed from the miRanda. Finally, the intersection of these two websites was taken as the final target genes of different expression miRNA.

Gene ontology (GO) and kyoto encyclopedia of genes and genomes (KEGG) analyses were carried out to further understand genes biological functions. Target genes of differential expressed miRNAs were mapped to GO terms in the database (http://www.geneontology.org/) and KEGG database (http://www.genome.jp/kegg/) respectively. The GO terms and KEGG pathways with *p*-value < 0.05 through hypergeometric test were defined as significantly enriched terms.

### Correlation analysis of miRNA and differentially expressed genes

In previous mRNA-seq studies, we have identified differentially expressed genes (DEGs) under heat stress using the same samples as the present study (Li, 2021). Therefore, correlation analysis of miRNA-mRNA were carried out in order to identify key miRNA-target pairs. *p* < 0.05 and fold change >2 were set as the threshold for screening miRNA-target pairs. Only inversely correlated miRNA-mRNA were identified. The identified miRNA-mRNA regulatory network was constructed by Cytoscape 2.8.3 software (Shannon et al., 2003).

### Quantitative real-time PCR validation of miRNA and mRNA expression

Quantitative real-time PCR (RT-qPCR) was used to verify the expression level of nine DEMs and four DEGs that were selected from miRNA-mRNA regulatory network based on their potential functional importance. The remaining RNA samples from the small RNA-Seq library construction were used for RT-qPCR amplifications. β-Actin and *U6* were used as the internal control gene for DEGs and DEMs (Table 1). Total RNA after genome DNA removing was reverse-transcribed to cDNA using Evo M-MLV RT Kit (TaKaRa, Japan) following the manufacturer’s instructions. The reaction was set for 25 min at 37°C and 5 s at 85°C. The cDNA was amplified using SYBR Green Premix Pro Taq HS qPCR Kit (TaKaRa, Japan) with miRNA and mRNA specific forward primers and universal reverse primers. The PCR amplification parameters were as follows: 95°C for 2 min, followed by 45 cycles of 95°C for 15 s, 60°C for 15 s, and 70°C for 25 s. Amplification specificity was verified by melting curve analysis. The relative expression levels of target gene transcripts were calculated according to the comparative cycle threshold (Ct) method (2^−ΔΔCT^), and GraphPad 6.0 was used for statistical analysis. All data are expressed as mean ± standard deviation (SD).

**TABLE 1:** RT-qPCR primers used for validation of this study.

Gene ID	Primer sequences (5′–3′)
miR-210	TAT​ACT​TGT​GCG​TGC​GAC​AGC​G
miR-31ac	AGG​CAA​GAT​GTT​GGC​ATA​GCT​GT
miR-10	CGC​TAC​CCT​GTA​GAT​CCG​AAT​TTG​TG
miR-2004-5p	GGC​TTT​CTG​TGG​CTG​TCG​TGT​TAA​G
miR-92a-3p	CTA​TTG​CAC​TTG​TCC​CGG​CCT​AT
miR-92a-5p	TAT​TGC​ACA​TGT​CCC​GGC​CTG
miR-92c	TTA​TTG​CAC​TCG​TCC​CGG​CC
miR-193	TAC​TGG​CCA​GCA​CAA​TCC​CAG
miR-4185	CGC​GTT​GTA​TTC​GTA​CTG​TCT​GAC​C
BAG3 R	GTT​ATC​GCC​TCT​CGG​TTC​AC
BAG3 F	GAC​TCT​CAG​CAT​CCA​TTC​TTC​A
WDR20 R	GGA​CTT​GAC​CAG​CCG​AGA​A
WDR20 F	AGC​AGC​AAT​TTA​ACA​CCA​GAG​A
TRAF7 R	ACA​GCA​GGA​GTA​TGA​CAA​GTG​A
TRAF7 F	GGA​ATG​GAA​TGA​AGC​GGT​TGA
FUT4 R	CGG​TCC​ACA​AGT​AGA​AGT​ACG
FUT4 F	TGG​CTT​AGG​TCG​CTC​TAT​GAT​A
ACTB R	GAT​GTC​ACG​GAC​GAT​TTC​ACG
ACTB F	AAG​GTT​ATG​CTC​TTC​CTC​ACG​CT
U6 R	TGG​AAC​GCT​TCA​CGA​ATT​TGC​G
U6 F	GGA​ACG​ATA​CAG​AGA​AGA​TTA​GC

## Results

### Small RNA library construction

In total, six small RNA libraries (CS1, CS2, CS3, HS1, HS2, HS3) were constructed from the body wall of sea cucumbers in the present study. The raw data have been submitted to the National Center for Biotechnology Information under the accession number SRR18918495 ∼ SRR18918500. A total of 11,929,100 and 10,107,557 raw reads were generated from the CS and HS, respectively. After removing low-quality reads, including repeat reads, junk reads, mRNA and other noncoding RNAs, a total of 5,902,588 and 5,053,528 valid reads were obtained from the CS and HS, respectively (Table 2). PCA analysis was performed on the data of six groups, and it was found that HS group and CS group had great repeatability, respectively (Supplementary Figure S1). From the valid reads length statistics, the distribution showed that 22 nt was the most common type (Supplementary Figure S2). A total of 403 known miRNAs were identified in six libraries (Supplementary Table S1). Among these known miRNAs, 281 miRNAs were grouped into 127 families, of which miR-25 was the largest family with 16 members, followed by let 7 family with 10 members, and the miR-1 with eight members (Supplementary Table S3). Among the 127 families, 61 families contained only one number, and 33 families contained two numbers. The information of top 20 families were displayed in a histogram (Supplementary Figure S3). The sequences nonmatched to miRBase database were compared with the genome to predict the novel miRNAs by miRDeep2 and 75 novel candidate miRNAs were predicted (Supplementary Table S4). Some novel miRNAs with the same mature sequence but different precursors were considered to belong to a novel miRNA family. These novel miRNA candidates were named in the form of “PC plus number” (Supplementary Table S4). The miRNA precursor sequences plus a sequence of 100 nt per site were presented in Supplementary Table S5.

**TABLE 2:** Statistics of small RNA sequences from the six libraries in the body wall of *Apostichopus japonicas*.

Types	CS1	CS2	CS3	HS1	HS2	HS3
Raw reads	11,070,085	11,460,240	11,543,525	12,595,348	12,071,954	11,336,397
3ADT and length filter	5,147,178	3,551,267	4,822,194	4,836,882	3,588,507	4,170,684
Junk reads	15,992	7,360	7,356	7,965	5,557	6,489
rRNA	371,333	157,862	358,843	495,445	209,038	124,374
tRNA	47,955	53,999	122,106	93,783	51,361	56,706
snoRNA	3,600	9,253	8,105	3,609	11,341	4,357
snRNA	3,295	2,589	7,585	3,236	2,714	2,331
mRNA	1,759,941	1,867,237	1,034,122	1,238,688	2,096,006	2,023,885
Repeats	51,088	36,052	66,679	43,662	50,584	17,811
Valid reads	3,659,175	5,772,797	5,109,872	5,849,747	6,052,171	4,927,402
Known miRNA	266	244	280	147	168	249
Novel miRNA	55	23	17	17	20	14

### Differential expression analysis of miRNAs

**FIGURE 1:**
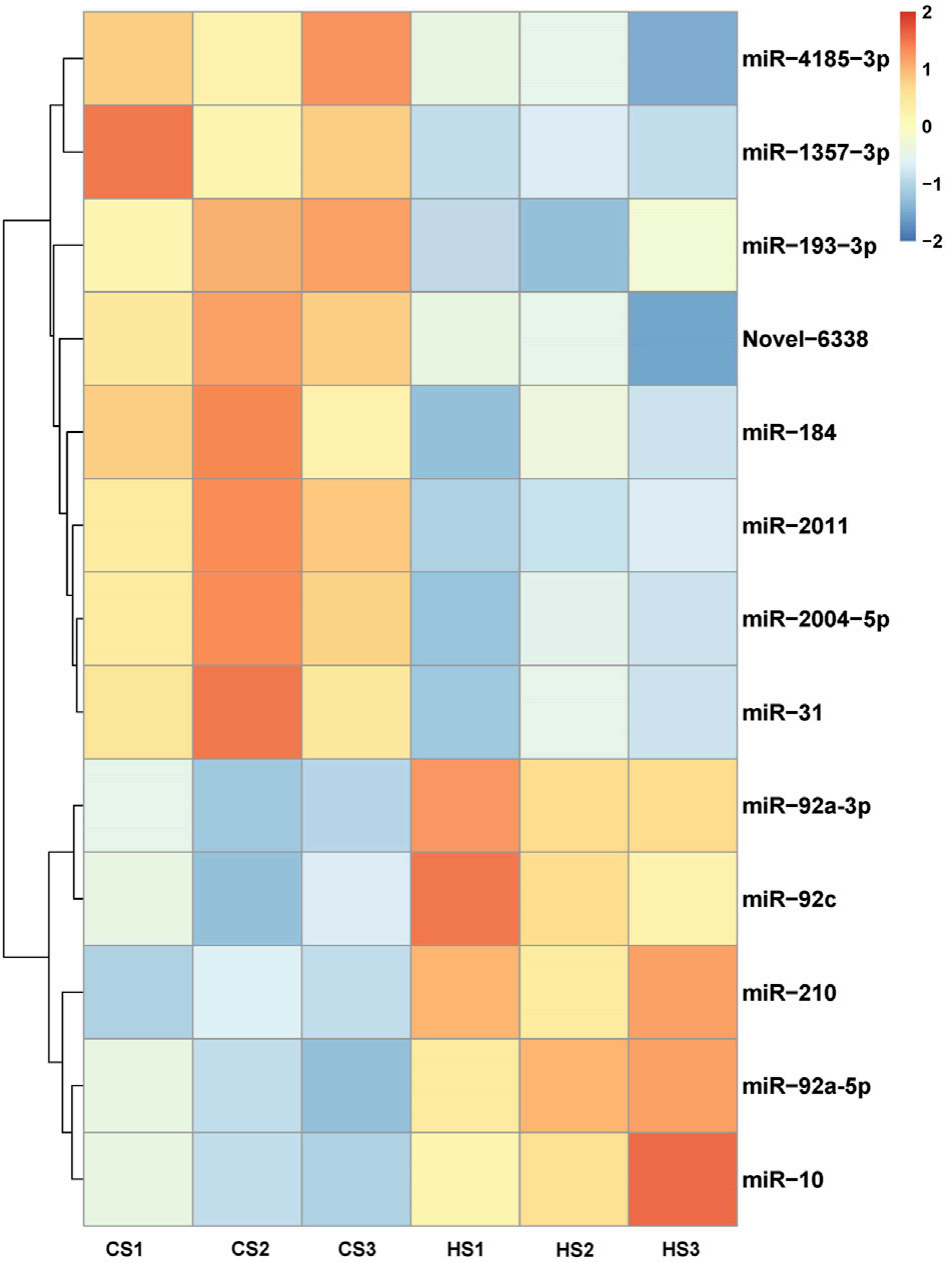
Cluster analysis heatmap of differentially expressed miRNAs.

Compared with the control group, 144 miRNAs were upregulated including miR-92a and miR-210, while 334 miRNAs were downregulated including miR-152 and miR-22. The *t*-test was used for analysis and *p* < 0.05 was regarded as the criterion to identify the differential expressed miRNAs (DEMs). The result showed that 13 DEMs were identified between CS and HS, and the numbers of upregulated and downregulated DEMs were five and eight, respectively (Figure 1; Table 3). These miRNAs may play an important role in regulating sea cucumbers in response to heat stress. Besides, there was a novel miRNA (Novel-6338) that included in these DEMs. Among these DEMs, the expression of miR-92a-3p and miR-210 showed more than two-fold up regulation and miR-10 showed more than five-fold up regulation. Particularly, the expression levels of miR-31, miR-2004-5p and miR-1357-3p were downregulated over four fold.

**TABLE 3:** Information of significantly differential expressed miRNAs in the body wall of *Apostichopus japonicas* under heat stress.

miRNA ID	Expression level of CS (mean ± SD)	Expression level of HS (mean ± SD)	log_2_ (fold_change)	Up/down
miR-92a-5p	171,784.572 ± 0.03	326,057.996 ± 0.06	1.12	Up
miR-92a-3p	231.004 ± 0.05	504.888 ± 0.08	1.13	Up
miR-2004-5p	132.433 ± 0.07	23.616 ± 0.07	−2.49	Down
miR-2011	17,240.464 ± 0.05	7,210.385 ± 0.03	−1.26	Down
miR-210	407.597 ± 0.04	951.313 ± 0.04	1.22	Up
miR-184	257,392.996 ± 0.04	122,704.002 ± 0.06	−1.07	Down
miR-193-3p	770.017 ± 0.06	371.259 ± 0.13	−1.05	Down
miR-31	5,632.975 ± 0.08	1,503.164 ± 0.10	−1.91	Down
miR-4185-3p	26.704 ± 0.09	7.890 ± 0.06	−1.76	Down
miR-92c	6,493.253 ± 0.03	14,052.467 ± 0.08	1.11	Up
Novel-6338	20.839 ± 0.13	6.583 ± 0.01	−1.66	Down
miR-1357-3p	27.683 ± 0.10	1.259 ± 0.02	−4.46	Down
miR-10	15,749.221 ± 0.07	82,172.348 ± 0.09	2.38	Up

### Target gene prediction and functional analysis

**FIGURE 2:**
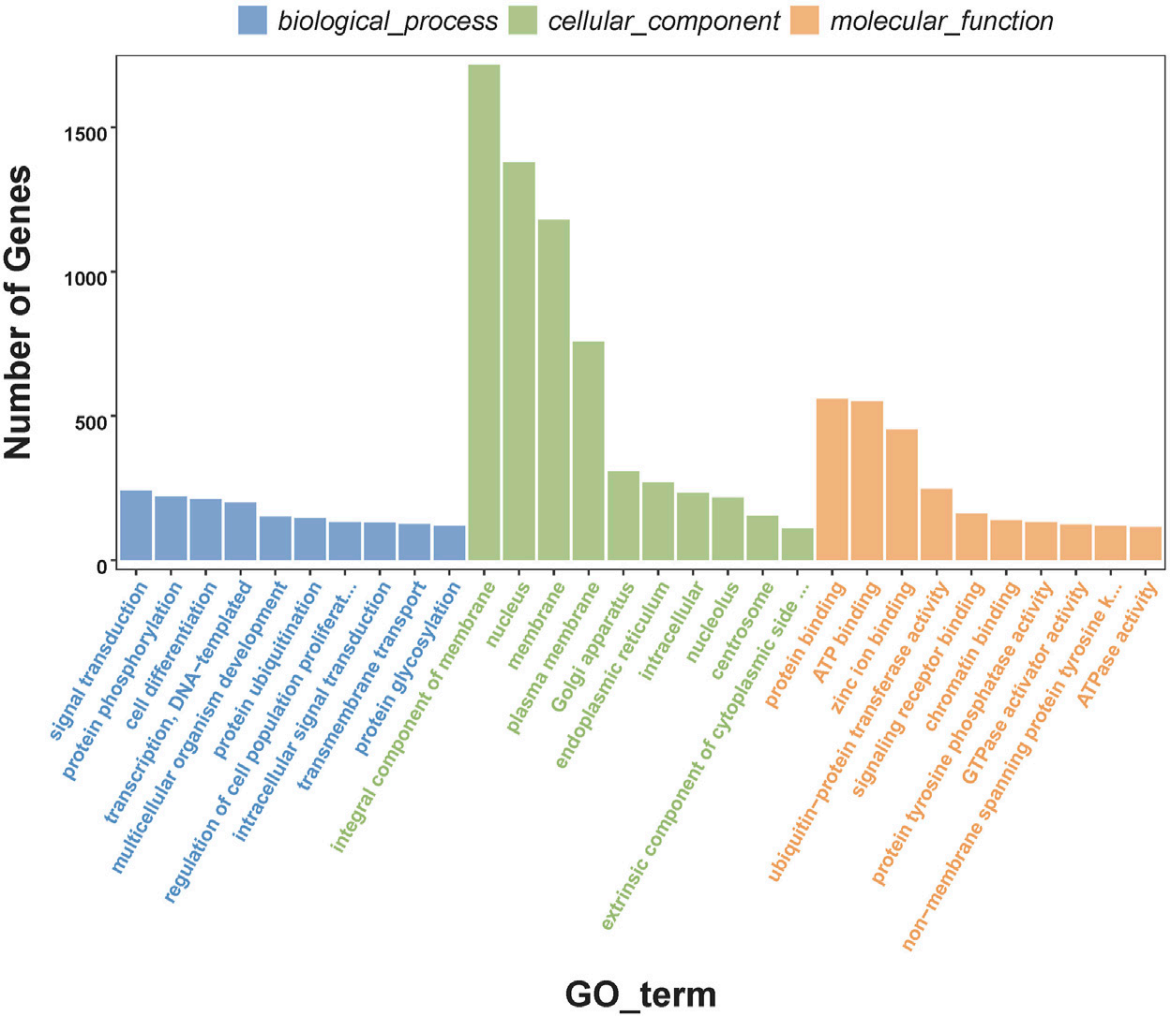
Top 10 enriched GO terms of the target genes of differentially expressed miRNAs.

**FIGURE 3:**
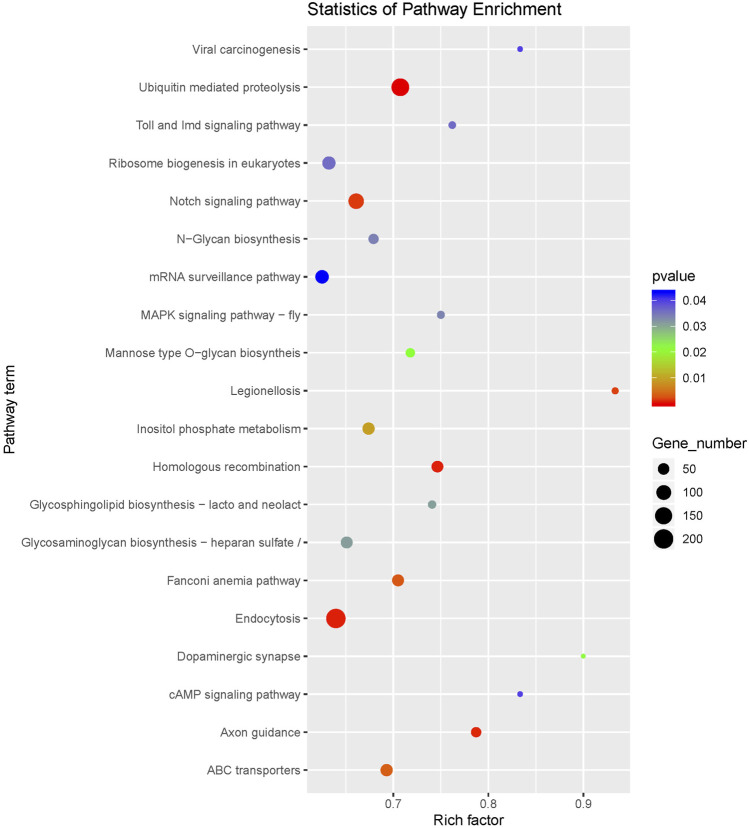
KEGG pathway enrichment of the target genes of differentially expressed miRNAs.

In order to understand the biological processes and molecular functions of miRNAs during heat stress, Targetscan and miRanda were used to predict the target genes of the 13 DEMs. TargetScan algorithm removes the target gene whose context score percentile is less than 50, and miRanda algorithm removes the target gene whose maximum energy is greater than −10. Finally, the intersection of these two softwares is taken as the final target gene of DEMs. For the 13 DEMs, a total of 16,563 target genes were predicted, including 5,153 target genes of five upregulated DEMs and 11,410 target genes of eight downregulated DEMs. The GO database annotation results showed that 11,001 target genes of 13 DEMs were annotated to 334 GO terms (*p* < 0.05), including 178 terms for biological processes, 65 terms for cellular components, and 91 terms for molecular functions. Of these GO terms, membrane, integral component of membrane and nucleus in cellular components are enriched with more genes, and we listed the top 10 terms with the most target genes number in each category (Figure 2). The result showed that 4,235 target genes were annotated into 25 KEGG pathways (*p* < 0.05), among which the endocytosis, ubiquitin mediated proteolysis, homologous recombination, axon guidance, notch signaling pathway interaction were the top five pathways with the most abundant target genes (Figure 3).

### Correlation analysis of miRNA-mRNA

**FIGURE 4:**
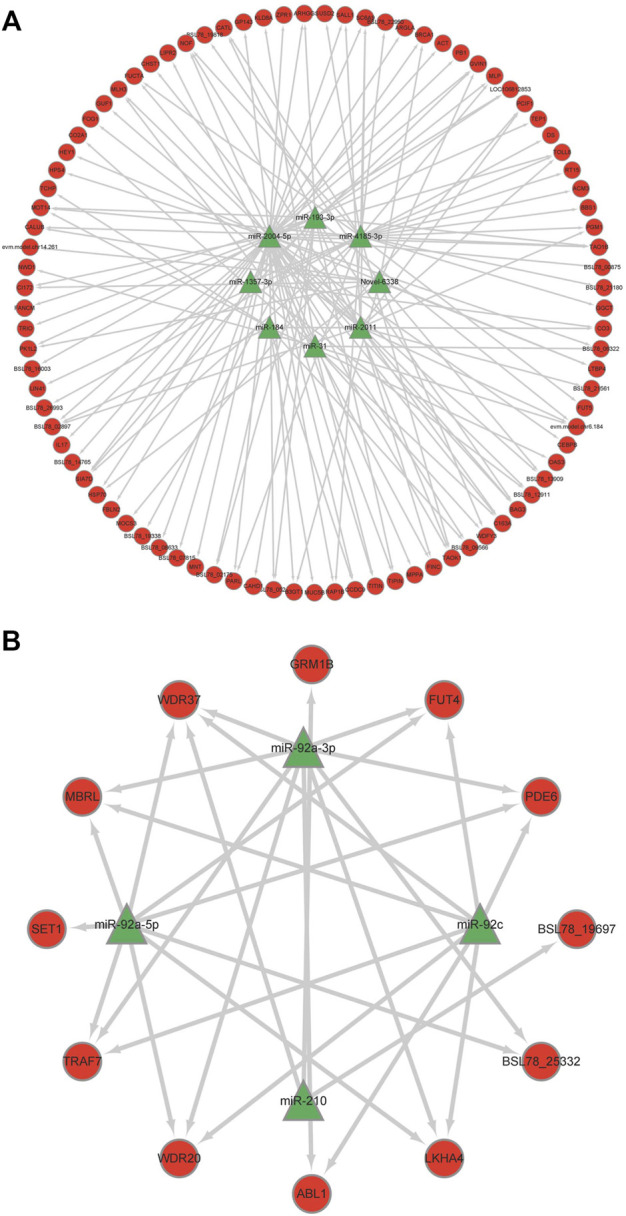
Regulation networks of miRNA-mRNA of key genes of correlation analysis. **(A)** Down-up mode; **(B)** Up-down mode.

To reveal the roles of miRNAs, the DEMs were selected for association analysis with the DEGs obtained by previously transcriptome sequencing. Finally, we identified 101 inversely correlated target genes that potentially regulated by miRNAs in response to heat stress of sea cucumbers (Supplementary Table S6). Of these 101 inversely correlated target genes, 12 inversely correlated target genes were down regulated by four up regulated DEMs, and 89 inversely correlated target genes were up regulated by eight down regulated DEMs. Among them, miR-92c combined with the most DEGs in the upregulated DEMs, and miR-2004-5p combined with the most DEGs in the downregulated DEMs (Figures 4A,B). The DEGs in the regulation network, such as WD repeat-containing protein 20 (WD20), glycoprotein 3-alpha-L-fucosyltransferase A-like (FUT4), E3 ubiquitin-protein ligase (TRAF), Serine/threonine protein kinase TAO3-like isoform (PAK4), interleukin -17D-like (IN17) and BAG family molecular chaperone regulator 3 (BAG3) involved in ubiquitin-mediated proteolysis and notch signaling pathway. These results suggested that these negative regulatory genes might be involved in the response of *A. japonicus* to heat stress.

### Validation of differentially expressed miRNAs and their differentially expressed genes expression by real-time PCR

**FIGURE 5:**
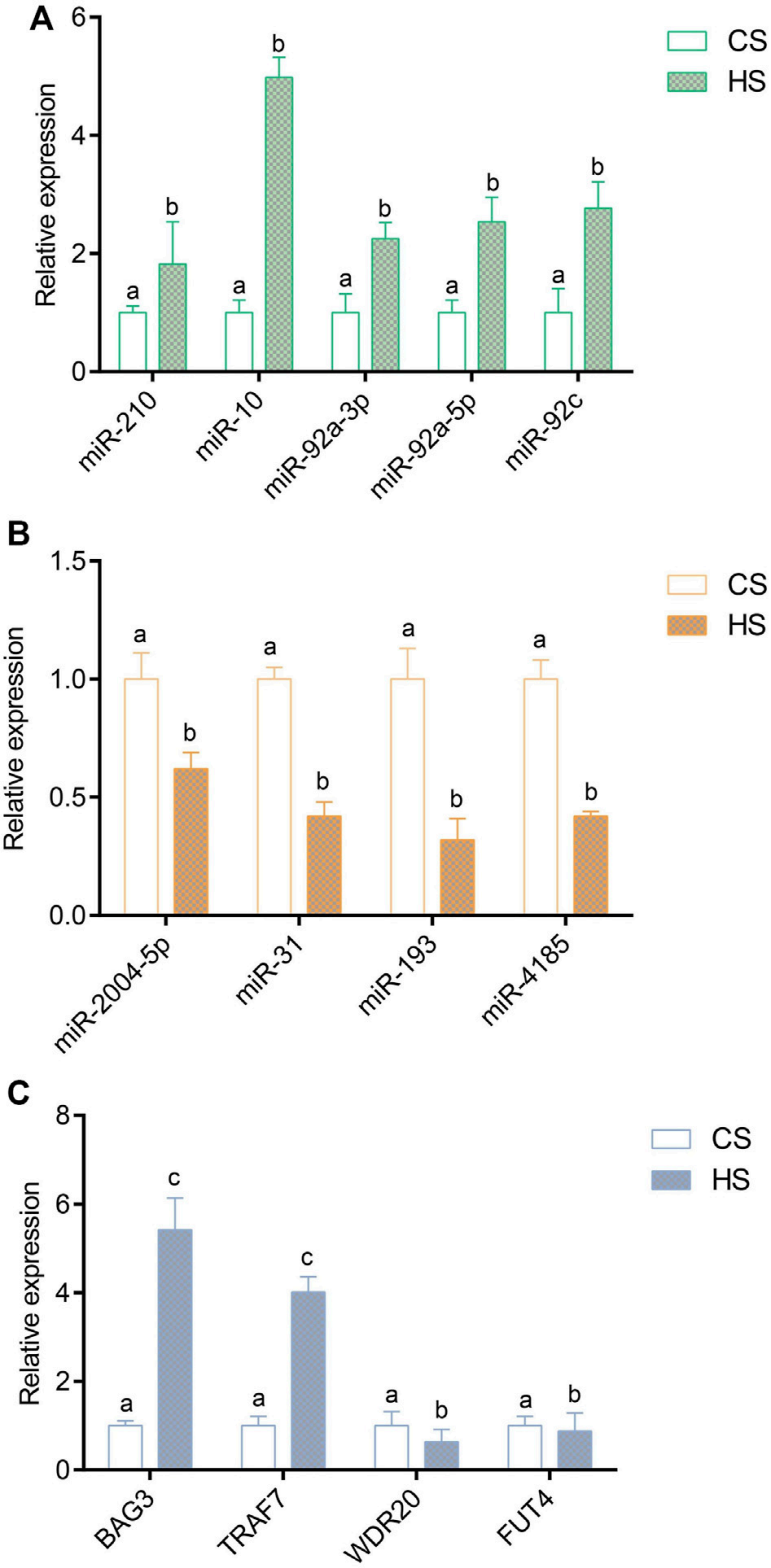
Validation of differentially expressed miRNAs and target genes *via* RT-qPCR method; **(A)** Validation of upregulated differentially expressed miRNAs; **(B)** Validation of downregulated differentially expressed target genes; **(C)** Validation of negative DEGs. Values indicate the mean ± SD (*n* = 3).

To verify the reliability of the sequencing expression profiles, nine miRNAs and four negative genes were applied to RT-qPCR. A peak was detected in the melting curve during the experiment, indicating that all PCR products were specifically amplified. The results of RT-qPCR showed that the expression trend of miRNA and negative genes were consistent with the high-throughput sequencing results. The expression of miR-210 and miR-10, miR-92a-3p, miR-92a-5p and miR-92c were significantly upregulated (Figure 5A), and miR-2004-5p, miR-31, miR-193 and miR-4185 were significantly downregulated (Figure 5B). The expression of BAG3 and TARF7 were significantly upregulated, and WDR20 and FUT4 were significantly downregulated (Figure 5C).

## Discussion

In the present study, we performed a high-throughput miRNA sequencing and miRNA-mRNA correlation analysis to study the post-transcription regulation mechanism of *A. japonicus* in response to high-temperature stress. We obtained 13 significantly differentially expressed miRNAs and a list of negative regulator genes of these differentially expressed miRNAs in the body wall of *A. japonicus* during heat stress. These miRNAs, negative target genes and miRNAs-mRNA regulation networks may play important roles in regulating heat stress, which broadens our understanding of the molecular regulation mechanism of *A. japonicus* in response to heat stress.

Among the 13 identified differentially expressed miRNAs, some miRNAs (such as miR-184, miR-193, miR-2004, miR-31, miR-210 and miR-10) have been reported to be involved in stress responses or immune processes previously. MiR-184 has been reported to be related to the infection of *Procambarus clarkii* by *Spiroplasma eriocheiris* (Ou et al., 2013). Xu et al. (2019) found that miR-184 of *Sinopotamon henanense* was significantly upregulated in response to oxidative stress induced by cadmium (Cd). MiR-193 has been reported to be related to *Bos taurus* infection (Shi et al., 2014). The expression level of miR-2004 was increased with the increase of *A. japonicus* complement *AJC3* in the coelomic cells of *A. japonicus* in different periods after LPS stimulation (Zhong et al., 2015). The results of Zhou et al. (2018) showed that miR-2004 was regarded as a significantly expressed gene in the coelomic cells of *A. japonicus* under the infection of *V. splendidus*. MiR-31 has been proved to regulate the occurrence of inflammatory bowel disease by directly targeting the hypoxia-inducible factor (Olaru et al., 2015). Suzarez et al. (2010) confirmed that miR-31 could regulate the inflammatory response by negatively regulating the binding of e-selectin and neutrophils to endothelial cells. Kang et al. (2019) found that miR-31 was significantly upregulated in the liver after infecting grouper with *V. alginolyticus*, and the target genes were mainly concentrated in immune-related pathways. Therefore, we inferred that miRNA plays a complex regulatory role in various stress responses.

MiR-210 was known as a major hypoxia-inducible microRNA, which is evolutionarily conserved and ubiquitously expressed in the hypoxic cell and tissue types, serving versatile functions (Chang and Loscalzo, 2010). It was found that miR-210 can negatively regulate the production of LPS-induced pro-inflammatory cytokines through NF-κB1 in mouse macrophages (Qi et al., 2012). Yu et al. (2021) found that miR-210 can activate PI3K/AKT pathway by regulating PI3K and p-AKT protein, promoting the proliferation, and inhibit the apoptosis of dental pulp stem cells. Recently, Li et al. (2016b) found that miR-210 regulated the host defense of *A. japonicus* through TLRs (Toll-like receptors) pathway. In the present study, the significantly upregulated expression indicates that miR-210 also play role in sea cucumber’s body wall in response to heat stress. It was speculated that *A. japonicus* may reduce the expression of target genes by upregulating the expression level of miR-210 to cope with the harm of heat stress.

MiR-10 family members were highly conservative and closely associated with metabolizing response. MiR-10 can directly regulate the expression levels of FLT1 (vascular endothelial growth factor receptor 1) and sFLT1 (soluble vascular endothelial growth factor receptor 1), both of which are closely related to the growth of vascular endothelial cells (Livak and Schmittgen, 2013). Knock-out of miR-10 could lead to premature termination of the growth of blood vessels in the embryonic internodes of zebrafish larvae, while over-expression of miR-10 could accelerate the angiogenesis in zebrafish and the growth of human umbilical vein endothelial cells (Wienholds, 2005). Another study, found that miR-10 can regulate the growth of blood vessel endothelial cells by promoting new signal transmission (Naguibneva et al., 2006). Overexpression of miR-10 could promote virus-induced apoptosis, and researchers discovered that the expression of apoptosis protein caspase-3 increased in tandem with miR-10 expression (Zhang, 2014). Moreover, miR-10 was significantly upregulated in the process of infection by infecting porcine alveolar macrophages with PRRSV (Porcine Reproductive and Respiratory Syndrome Virus), and the transfection experiment demonstrated that the upregulated expression of miR-10 could significantly inhibit the replication of *HP-PRRSV* and *N-PRRSV* in porcine alveolar macrophages (Zhao et al., 2017). In the present study, we found that miR-10 was significantly upregulated 5-fold after heat stress. It indicates that miR-10 regulates the metabolism of *A. japonicus* during heat stress, but it is molecular function needs further to be studied.

In the process of *A. japonicus* in response to heat stress, ubiquitin-mediated proteolysis and notch signaling pathway were significantly enriched, indicating that they played important role in the process. Ubiquitin-mediated proteolysis is an important cellular immune pathway, which can control the basic life activities of cells by degrading key regulatory proteins and regulating the cellular stress response and immune response to pathogenic microorganisms (Chen et al., 2012). For example, when shrimp was infected by *V. cholerae*, ubiquitin proteins can mediate cellular immunity to cope with the infection (Li, 2020). The notch signaling pathway can regulate cell differentiation, proliferation and apoptosis, which is of great significance to cell growth and development. Abnormal expression of notch signaling can induce many kinds of cancers, such as breast cancer (Wang et al., 2017), lung cancer (Tian et al., 2017), and gastric cancer (Wang et al., 2018). We inferred that ubiquitin-mediated proteolysis and Notch signaling pathway could resist the effects of high temperature by regulating the expression of downstream immune genes in the process of heat stress.

Through the correlation analysis between the differentially expressed miRNAs and transcriptome data, it found that 12 DEMs can target 101 inversely correlated DEGs. TRAF7 has been partially studied in aquatic animals, and BAG3, WDR20 and FUT4 are mostly focused on wound tissue recovery and cancer treatment in medicine. BAG3 is a member of the BAG gene family, which plays an important role in regulating cell apoptosis, autophagy, movement and development, and mediating the adaptability of cells response to heat stress (Rosati et al., 2011). The results of GO enrichment analysis showed that BAG3 could inhibit cell apoptosis and promote tumor cell proliferation by binding to HSP70 (Manzerra and Brown, 1990). TRAF7 belongs to the TRAFs family, which involves a variety of biological functions including innate immunity, embryo development, stress response and inflammatory response (Reuss et al., 2013). In mammals, TRAF7 can regulate the signal activities by enhancing MEKK (mitogen-activated protein kinase), and then participate in the immune response of the body (Xu et al., 2004). *Cynoglossus semilaevis* has also been found to downregulate TRAF7 expression in all tissues infected with *V. harveyi*, particularly the liver (Wei et al., 2018). Studies have shown that WDR20 has a positive or negative correlation with the occurrence of lymphoma, prostate cancer and other diseases (Ohashi et al., 2015). At the same time, experiments have also proved that miR-3188 can target and inhibit the expression of WDR20 (Zhang et al., 2018). Screening of key genes of *A. japonicus* in response to heat stress provides basic data for elucidating its regulation mechanism. Further research will focus on the verification of the regulatory relationship between DEMs and DEGs.

## Conclusion

In this study, we identified the expression profiles of miRNAs under heat stress in *A. japonicus* by using small RNA-seq. We highlighted 13 DEMs compared with the control group, which were involved of immunity response and cellular activity. In addition, we performed a correlation analysis of DEMs and differentially expressed genes in sea cucumber and 101 key negative regulator genes of DEMs were obtained, which may play important roles in regulating the process of *A. japonicus* under heat stress. Our study provided an increasing understanding of miRNAs’ roles in *A. japonicus* during heat stress. Through the discovery of their related targets, we have a deeper understanding of miRNAs and functional genes. In conclusion, our results provided new insights into the miRNA regulation and molecular adaptation mechanisms of *A. japonicus* under heat stress.

## Data Availability

The datasets presented in this study can be found in online repositories. The names of the repository/repositories and accession number(s) can be found below: Sequence read archive under the accession number SRR18918495 ∼ SRR18918500.

## Supplementary material

Supplementary Figure S1 PCA analysis of six body wall libraries in *Apostichopus japonicas*.

Supplementary Figure S2 Length distribution of valid reads in six libraries in the body wall of *Apostichopus japonicas*.

Supplementary Figure S3 Families and members distribution of known miRNAs.
