# Supplementary figures and images for: Differential expression of miRNAs in the body wall of the sea cucumber Apostichopus japonicus under heat stress
Source: Front Physiol. 2022 Jul 21;13:929094. doi: 10.3389/fphys.2022.929094 (PMC9351827; doi:10.3389/fphys.2022.929094)

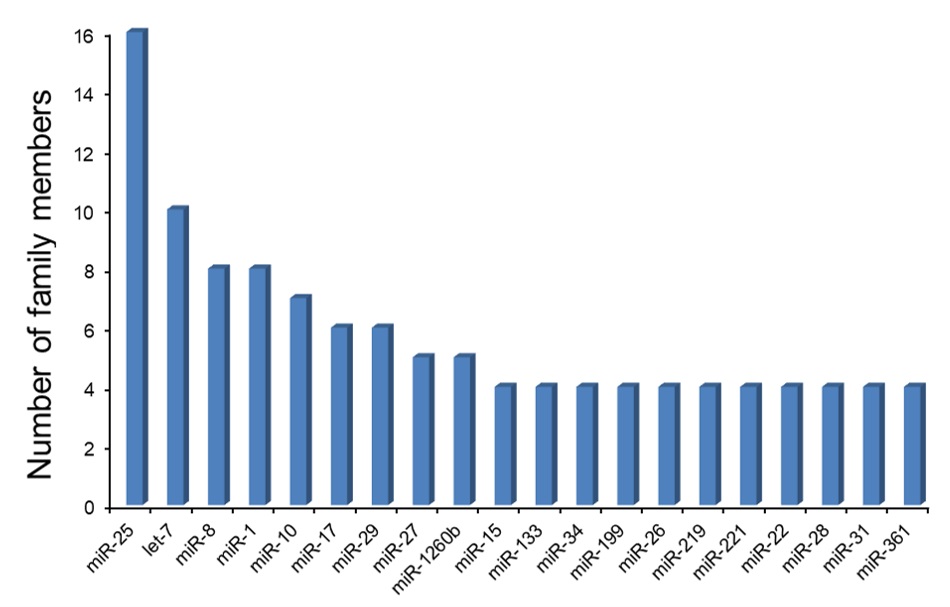

Supplement: Supplementary file 2 [file Image3.JPEG]

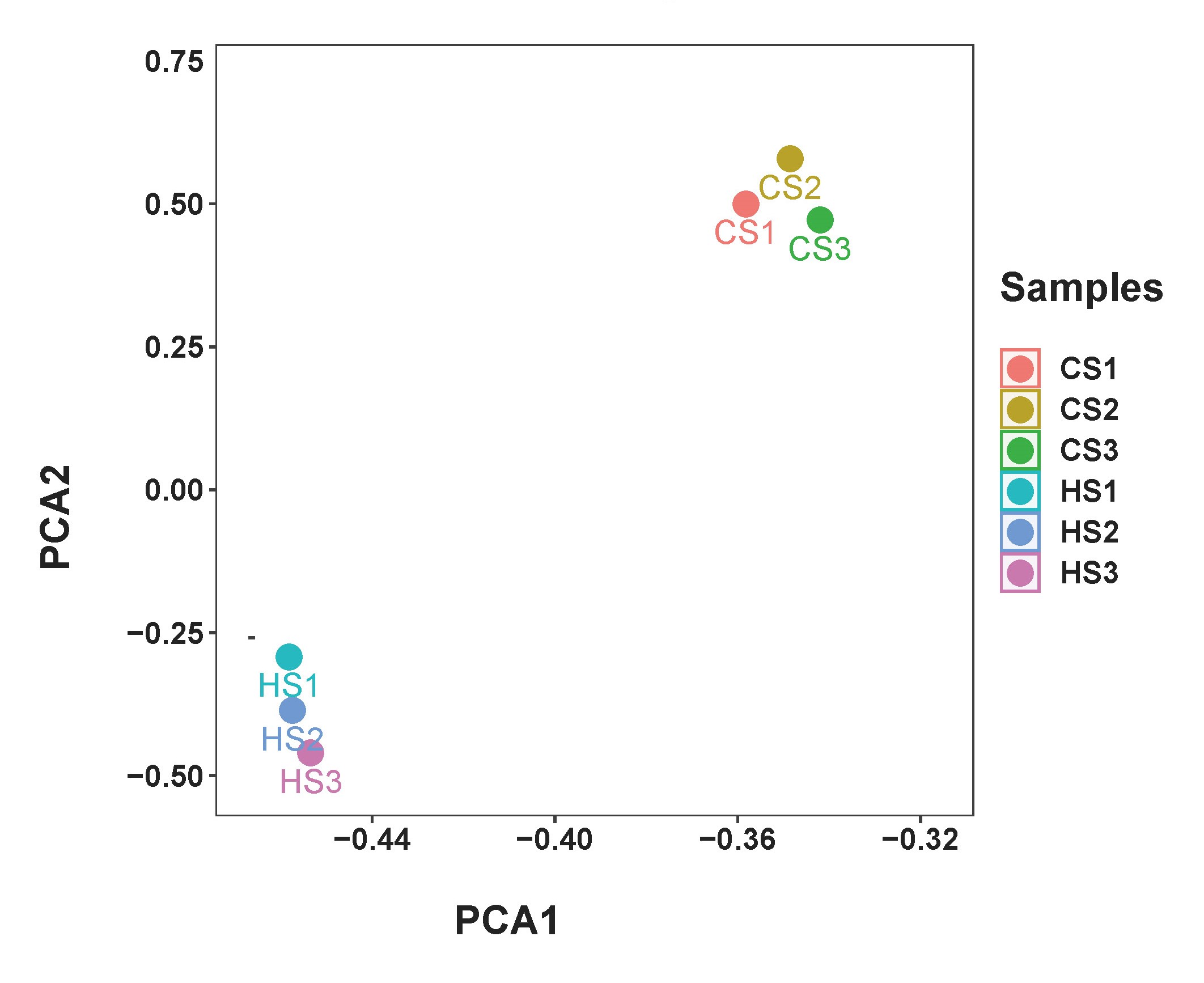

Supplement: Supplementary file 4 [file Image1.JPEG]

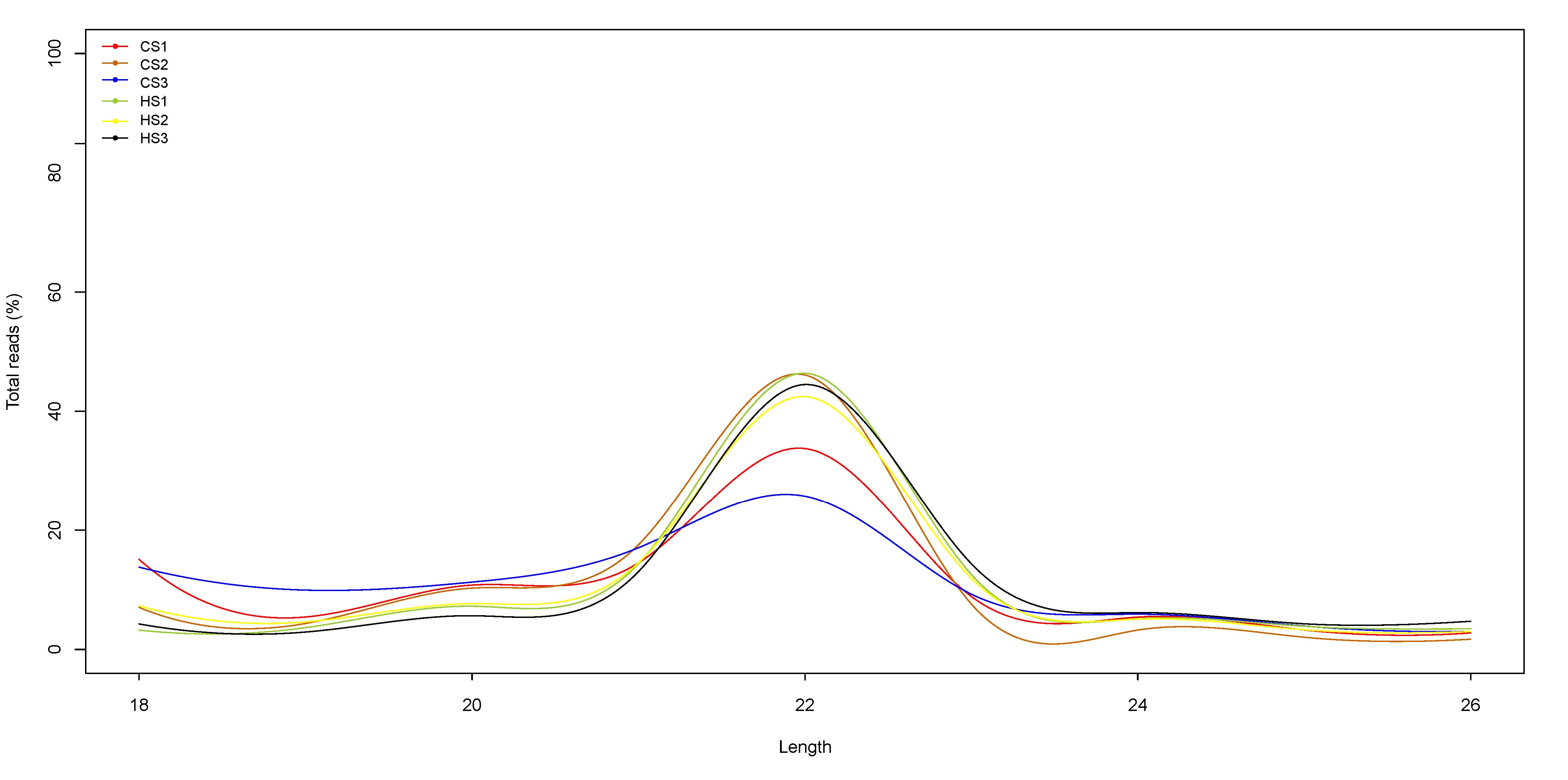

Supplement: Supplementary file 5 [file Image2.JPEG]
